# Estimation of the age of majority through radiographic evaluation of the third molar maturation degree

**DOI:** 10.4317/medoral.23385

**Published:** 2020-02-10

**Authors:** Miriam del Carmen Marrero-Ramos, Lourdes López-Urquía, Aldo Suárez-Soto, Almudena Sánchez-Villegas, Mario Vicente-Barrero

**Affiliations:** 1 MD. Dr Negrin University Hospital of Gran Canaria. Spain; 2 MD. Maxillofacial surgeon. Insular University Hospital of Gran Canaria. Spain; 3 MD, PhD. Professor in Preventive Medicine and Public Health. University of Las Palmas de Gran Canaria. Spain; 4 MD, DDS, PhD. Insular University Hospital of Gran Canaria. Assistant professor at the University of Las Palmas de Gran Canaria. Spain

## Abstract

**Background:**

Although the third molar is the tooth with the greatest anatomical and developmental variability, some authors consider it important to estimate whether a subject is of legal age or not. The Demirjian’s technique is the most widely used tool to estimate dental age and was therefore used in our study to assess possible correlation between the age of majority and the maturational degree of the lower third molars, evaluated through radiographic images.

**Material and Methods:**

This observational transversal study was conducted on 180 panoramic radiographs from consecutive patients. The degree of maturation of the lower third molar was independently classified by two observers, according to the Demirjian´s maturational stage method.

**Results:**

A total of 180 patients – 65 men (36.12%) and 115 women (63.88%) – were included (mean age 21.6 years; standard deviation 5.2). The age range of our subjects corresponded to maturational stages D to H in the Demirjian’s classification. A logistic regression analysis showed that subjects classified into the highest maturational stage H had a significantly higher probability of being considered of legal age by both observers, as compared with subjects in the lowest stage D. Inter-observer agreement was very high. Gender predictive capacity was not observed.

**Conclusions:**

Our results showed that subjects with the lower third molars in the highest maturational stage could be estimated to be older than 18 years and therefore considered of legal age, a finding also reported by other authorsThe Demirjian´s tooth maturational stage method, applied to the lower third molars, can be reliably used to estimate whether an individual is of legal age (18 years or older). High concordance between different observers using this method can be expected.

** Key words:**Age estimation, third molar development, panoramic radiographs, Demirjian’s method, dental age estimation, forensic odontology.

## Introduction

Nowadays, a number of available diagnostic methods can be used in forensic estimation of biological age, something that may be necessary in certain situations like illegal immigration, criminal responsibility, etc. (1,2). However, to estimate the age of live adolescents and young adults for medical-legal purposes, only some methods are considered to have an accepTable scientific basis, due to their relative accuracy and amount of supporting scientific studies conducted on different populations. In Pediatrics, the most widely used method is based on the maturation of the left hand carpal bones (3). Other indicators include the developmental stage of cervical vertebrae and skull (4) or the degree of calcification of the medial clavicular epiphysis (5).

Although biologically important, the above indicators have no legal validity, since they cannot be used to determine whether an individual has reached 18 years of age, i.e. the age of legal majority in many countries including Spain. However, age-estimation methods based on tooth formation stages seem to be more reliable than those based on skeletal development (6). Radiographic evaluation of the maturational degree of the third molar produces useful images and is a non-invasive method (although involved X-radiation exposure should be considered) (6). The Demirjian´s tooth development staging technique (7) is the most extensively used tool for age estimation through dental examination. In this study, we used the Demirjian´s technique to estimate subjects’ age through radiographic images of the third molars. Our objective was to assess the reliability of this method to estimate whether a subject has reached legal age.

## Material and Methods

This transversal observational study included 180 panoramic radiograph images corresponding to 180 consecutive patients, managed at the Service of Dentistry Oral and Maxillofacial Surgery Service of the Complejo Hospitalario Materno-Insular of Las Palmas de Gran Canaria, between January and July 2016. Subjects’ images, sex, date of birth and date of x-ray study were obtained from the Electronic Medical Record (DRAGO platform) of the Canarian Health Service. The included extra-oral panoramic x-ray images were made with a digital orthopantomography equipment SATELEC X-mind panoD+; software DIGORA DfW 2.5 R1 (Windows).

The inclusion criteria for subject selection were: patient or a legal guardian signing the provided informed consent form (anonymity was granted throughout the study), subject between 15 and 30 years old, subject with of both lower third molars (right and left), subject without third molar malformation or deformity.

This study was approved by the Hospital’s Commission for Research, Teaching and Training.

Images were assigned a code number, in order to hide patients’ age and sex, and were evaluated by two independent observers with the aim of reducing observer bias.

**Figure 1:**
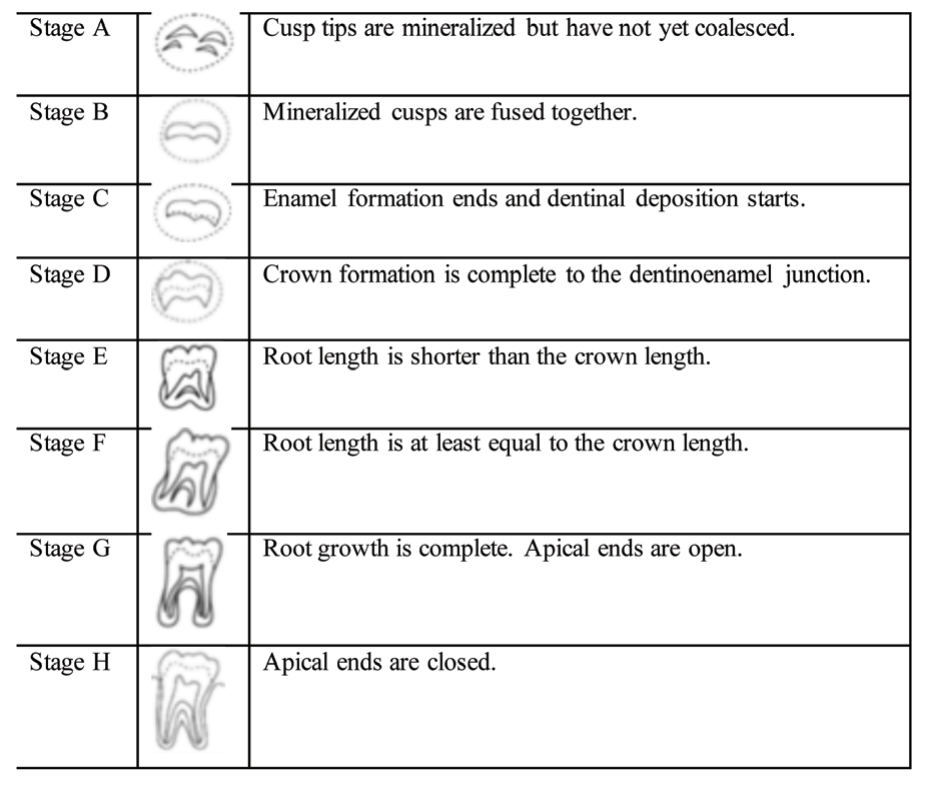
Demirjian’s tooth maturational stages for the third molar include 8 stages (described by Demirjian, modified by Kasper).

In a first session, both observers were trained by evaluating 25 radiographs and using the Demirjian´s method to classify subjects into different stages from “D” to “H”. The observers evaluated the maturational degree of both left and right lower third molars of each subject. Notice that the age range of patients included in this study corresponded to stages D, E, F, G, H of the Demirjian’s classification (from lowest to highest maturational stage). Fig. 1 shows Demirjian´s tooth maturational stages for the third molar include 8 stages (modified by Kasper) (8).

In a subsequent session (immediately after the first one), the observers evaluated the 180 radiographs corresponding to the selected patient sample (including the above mentioned 25 radiographs used for training). The observers independently estimated the Demirjian’s stage of the lower third molars for each subject, (notice that both molars in a subject were at the same maturational stage). Thus, this variable was recorded twice: once by observer 1 and once by observer 2. For the statistical analysis, categories D to H were assigned numerical values and entered into an Excel 2016 Table (Microsoft Office).

The chronological age of each subject on the day of the x-ray study was recorded in years (age was intentionally hidden from the observers).

The statistical analysis was conducted with the SPSS version 22 software package.

Logistic regression was used to evaluate the probability of subjects being of legal age (18 years or older) according to their molar maturational stage. Thus, the maturational degree was considered an ordinal qualitative variable and dummies were created (Table 1); this variable was dichotomous and every category was compared with the lowest-maturational stage D. On this basis, different analyses were carried out to evaluate the capacity of the third molar maturational stage to predict legal age, as well as to evaluate inter-observer differences. Odds ratios (OR) and confidence intervals were calculated for every observer.

**Table 1:**
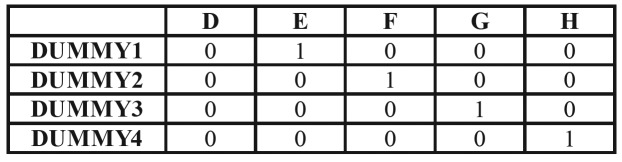
Four dummies were created for every observer.

## Results

The study included 180 patients, who fulfilled the inclusion criteria: 65 men (36.12%) and 115 women (63.88%); mean age 21.6 years (standard deviation 5.2).

Table 2 shows the maturational stages estimated by both observers independently. Noticeably, both of them estimated stage D with the lowest frequency (n=6 for observer 1 and n=5 for observer 2) and stage H with the highest one (n=94 for observer 1 and n=99 for observer 2).

Since between-sex differences were not found for the predictive capacity of the Demirjian’s method, analysis stratified by sex was not conducted.

**Table 2:**
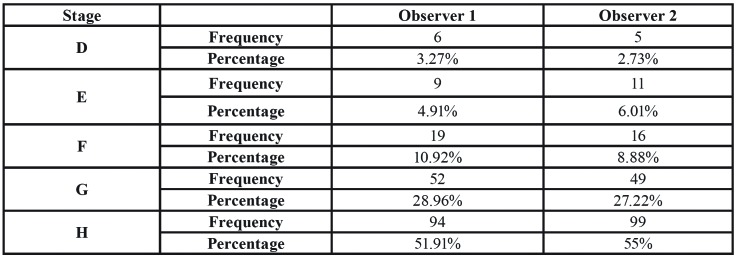
Frequencies of the different Demirjian’s stages, as estimated by every observer independently.

Table 3 shows the comparison of every maturational stage D to H versus the lowest maturational stage D, as evaluated by every observer; odds ratios (OR) and confidence intervals were calculated.

For observer 1, only the comparison “stage H” vs. “stage D” (i.e. the highest versus the lowest maturational stage) was statistically significant (OR=29.333). Namely, compared with subjects in stage D, subjects in stage H had a significant 25-times higher probability of being of legal age. Noticeably, results from observer 2 were rather similar; i.e. the highest maturational stage was associated with a significant about 20-times-higher probability of being of legal age than the lowest stage (OR=23.250).

The intra-class correlation coefficient and the percentage of wrong classification were calculated in order to assess inter-observer differences. Such analysis showed a very high degree of concordance between both observers; maximum value was 1 (Table 4).

The percentage of wrong classification was 13.7%. Since the wrong/right classification percentage could be partially random, we studied the weighted Kappa index of agreement, where random effects were eliminated. The kappa index was 0.781, which was considered to reflect a high degree of agreement.

**Table 3:**
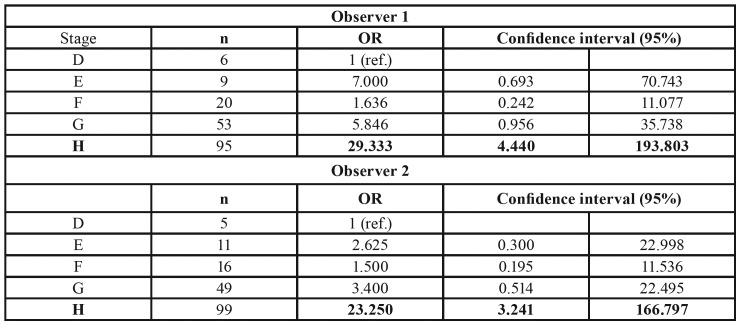
Comparison of every maturational stage D to H versus the lowest maturational stage D, as evaluated by every observer.

**Table 4:**
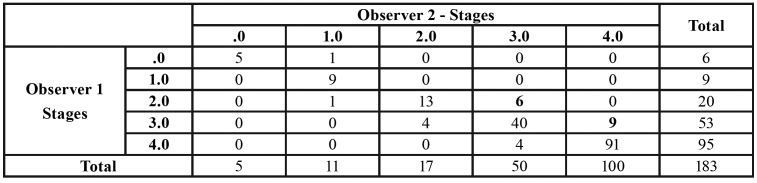
Degree of concordance between both observers.

## Discussion

In legal terms, the chronological age is the amount of time a person lived since birth. The consequences of not knowing this amount of time exactly may be serious for a person and/or a country, or even be the cause of an international conflict. Surprisingly, cases of unknown chronological age are increasingly frequent in developing countries, which makes it necessary to develop reliable age-estimation methods thus posing a challenge to science. As an example, forensic estimation of age is crucial in establishing criminal responsibility, which depends on whether the involved subject has reached the age of majority or not. Similarly, the complexity of estimating the age of undocumented young adults and its consequences need to be acknowledged (9).

In our opinion, assessing the maturational degree of the third molar should be the method of choice, when a radiographic approach is used, because it is not invasive and produces images easily and quickly (6). Main advantages of usign orthopantomography include: wide anatomical coverage, relatively low radiation exposure, convenience, ease and speed of the procedure. Disadvantages include: lack of detail, lack of comparison with intraoral images, overprojection of anatomical structures, variable amplification and geometric distortion inherent to image generation (9).

It should be taken into account that all the included orthopantomographs were made for reasons other than the aim of the study (for example, tooth extraction); namely, our subjects were not exposed to radiation for the purpose of the study.

The third molar is the tooth with the most variable anatomy and development; some authors even consider it unpredicTable (2,10). The Demirjian’s technique is the most extensively used validated tool to estimate dental age; therefore, we used it to evaluate the third molar maturity and its role in the estimation of legal age. To that end, we assessed the possible correlation between the age of majority and the degree of calcification of the third molar as evaluated through radiographic images.

It should be taken into account that age estimation is imprecise and inaccurate. It is not possible to know an individual’s exact age and considerable errors of 20-24 months have been reported. Thus, the choice of an age-estimation method usually depends on the experience of the researcher. In this regard, tooth formation is a suiTable tool for age estimation, since it is a progressive and continuous process,which can be entirely followed through x-ray images, from the stage of crypt formation to the closing of the root apex. Additionally, once a tooth is completely mineralized and emerged, it is a relatively sTable entity, formed by strong tissue that resists post-mortem degradation. Third molars have the unique advantage that their development continues over a long period, until older ages (6).

Almost all the studies aimed at validating different methods of dental age estimation involve the method developed by Demirjian *et al*., including some studies conducted in Spain (9,11). Although there are other methods (12,13), in general, this one is widely accepted (3). Demirjian *et al*. proposed their method of dental age estimation based on the chronology of teeth mineralization, in 1973 (7,14).

Given the important consequences of establishing whether an individual is of legal age or not, all available data should be taken into account in the process of age estimation, e.g. physical examination and radiographic study of the left hand and the teeth, in order to make a decision (10,11).

In our logistic regression study, we calculated the probability of a subject being of legal age (18 years or older) according to the maturational stage of the third molars. Results indicated that subjects classified in stage H of the Demirjian’s classification could be estimated to be older than 18 years and therefore considered of legal age; this result was statistically significant. This finding was comparable to the results of reported by Mincer *et al*. (15) in a study conducted on 823 subjects of 14 to 24.9 years of age, in USA and Canada. The authors concluded that only stage H was predictive of legal age. Similarly, our study showed 95% confidence intervals and ORs of 29.333 and 23.250 for observer 1 and observer 2, respectively (Table 3).

Regarding differences between both sexes, several authors reported that certain stages of the third molar development occur earlier in men than in women (8,14-17), thus providing evidence that the developmental degree of this tooth is higher in males. It should be noticed that this finding only applies to the third molar, since the development of all the other permanent teeth occurs earlier in women (1,2). In our study however, the effect of this variable could not be adequately evaluated because the distribution per sex was not homogeneous; thus, results were estimated for both sexes together.

We would like to highlight that intra- and inter-observer reliability (Table 4) was rather high, so that it was unlikely to bias the results of our study and their interpretation. This finding was similar to those of other studies involving more than one observer (2).

## Conclusions

From the analysis of our results, we concluded that the Demirjian´s tooth maturational stage method, applied to the lower third molars, can be reliably used to estimate whether an individual is of legal age (18 years or older), and that high concordance between different observers can be expected.
